# Effect of transcranial static magnetic stimulation over unilateral or bilateral motor association cortex on performance of simple and choice reaction time tasks

**DOI:** 10.3389/fnhum.2023.1298761

**Published:** 2023-12-04

**Authors:** Takuya Matsumoto, Tatsunori Watanabe, Kanami Ito, Takayuki Horinouchi, Sumiya Shibata, Hiroshi Kurumadani, Toru Sunagawa, Tatsuya Mima, Hikari Kirimoto

**Affiliations:** ^1^Faculty of Health Sciences, Tokyo Kasei University, Saitama, Japan; ^2^Faculty of Health Sciences, Aomori University of Health and Welfare, Aomori, Japan; ^3^Sakamoto Hospital, Osaka, Japan; ^4^Department of Sensorimotor Neuroscience, Graduate School of Biomedical and Health Sciences, Hiroshima University, Hiroshima, Japan; ^5^Japan Society for the Promotion of Science, Tokyo, Japan; ^6^Department of Physical Therapy, Niigata University of Health and Welfare, Niigata, Japan; ^7^Institute for Human Movement and Medical Sciences, Niigata University of Health and Welfare, Niigata, Japan; ^8^Department of Analysis and Control of Upper Extremity Function, Graduate School of Biomedical and Health Sciences, Hiroshima University, Hiroshima, Japan; ^9^Graduate School of Core Ethics and Frontier Sciences, Ritsumeikan University, Kyoto, Japan

**Keywords:** transcranial static magnetic stimulation, non-invasive brain stimulation, premotor cortex, simple reaction time task, choice reaction time task, motor association cortex

## Abstract

**Background:**

Transcranial static magnetic stimulation (tSMS) is a non-invasive brain stimulation technique that place a strong neodymium magnet on scalp to reduce cortical excitability. We have recently developed a new tSMS device with three magnets placed close to each other (triple tSMS) and confirmed that this new device can produce a stronger and broader static magnetic field than the conventional single tSMS. The aim of the present study was to investigate the effect of the conventional single tSMS as well as triple tSMS over the unilateral or bilateral motor association cortex (MAC) on simple and choice reaction time (SRT and CRT) task performance.

**Methods:**

There were two experiments: one involved the conventional tSMS, and the other involved the triple tSMS. In both experiments, right-handed healthy participants received each of the following stimulations for 20 min on different days: tSMS over the unilateral (left) MAC, tSMS over the bilateral MAC, and sham stimulation. The center of the stimulation device was set at the premotor cortex. The participants performed SRT and CRT tasks before, immediately after, and 15 min after the stimulation (Pre, Post 0, and Post 15). We evaluated RT, standard deviation (SD) of RT, and accuracy (error rate). Simulation was also performed to determine the spatial distribution of magnetic field induced by tSMS over the bilateral MAC.

**Results:**

The spatial distribution of induced magnetic field was centered around the PMd for both tSMS systems, and the magnetic field reached multiple regions of the MAC as well as the sensorimotor cortices for triple tSMS. SD of CRT was significantly larger at Post 0 as compared to Pre when triple tSMS was applied to the bilateral MAC. No significant findings were noted for the other conditions or variables.

**Discussion:**

We found that single tSMS over the unilateral or bilateral MAC did not affect performance of RT tasks, whereas triple tSMS over the bilateral MAC but not over the unilateral MAC increased variability of CRT. Our finding suggests that RT task performance can be modulated using triple tSMS.

## 1 Introduction

Transcranial static magnetic stimulation (tSMS) now has become a new member of non-invasive brain stimulation (NIBS). TSMS can reduce cortical excitability by placing a strong neodymium, iron, and boron (NdFeB) magnet that generates moderate-intensity (about 500 mT) static magnetic field (SMF) on scalp (Oliviero et al., 2011). In comparison to the other NIBS expected to induce inhibitory effects, such as cathodal transcranial direct current stimulation (tDCS) (Nitsche and Paulus, 2000), low-frequency repetitive transcranial magnetic stimulation (LF-rTMS) (Chen et al., 1997), continuous theta-burst stimulation (cTBS) (Huang et al., 2005), which induce electric current flow, tSMS (that induces SMF) causes less discomfort to the participants and is safe, economical, and easy to handle. In the past decade, various local brain regions such as the sensorimotor (Silbert et al., 2013; Kirimoto et al., 2014, 2016, 2018; Nojima et al., 2015, 2019; Davila-Perez et al., 2019; Nakagawa et al., 2019; Shibata et al., 2020), supplementary motor (Kirimoto et al., 2016; Pineda-Pardo et al., 2019; Tsuru et al., 2020; Guida et al., 2023), visual (Gonzalez-Rosa et al., 2015; Oliviero et al., 2015; Lozano-Soto et al., 2018), and dorsolateral prefrontal (Sheffield et al., 2019; Chen et al., 2021; Watanabe et al., 2021, 2023; Soto-León et al., 2023) cortices have been revealed to be modulated by tSMS, with potential clinical applications for neurological disorders (Di Lazzaro et al., 2021; Dileone et al., 2022; Shimomura et al., 2023). In addition, a new tSMS device constructed with three NdFeB magnets (called “SHIN jiba”) was introduced last year, and simulation has revealed that this triple tSMS can produce the greater static magnetic fields than the conventional tSMS (Shibata et al., 2022). However, its effect on behavioral performance has not been clear to date.

Anatomical and neurophysiological studies using monkeys showed that the dorsal premotor cortex (PMd) is involved in selection and planning of visually guided motor action (Mushiake et al., 1991). Also, human studies have demonstrated the importance of the PMd in action selection to visual cues, with the left hemisphere exhibiting dominance in rapid action selection (Schluter et al., 1998). In addition, recent functional magnetic resonance imaging (fMRI) research has revealed that the left PMd is engaged in all processes of visuomotor task, whereas the right PMd specifically contributes to rule-based visuomotor control and action preparation (Nakayama et al., 2022). Based on these findings, previous studies examining the effect of NIBS on the PMd in healthy individuals have evaluated performance of visual reaction time (RT) tasks. So far, ones that examined the effect of inhibitory NIBS over the PMd using these tasks have reported inconsistent results: some reported declines in the performance (Schlaghecken et al., 2003; Mochizuki et al., 2005; Gorbet and Staines, 2011), while the others reported no changes in the performance (O’Shea et al., 2007; Ward et al., 2010; Lu et al., 2012). The lack of inhibitory effects found in the later studies may be ascribed to a compensation within the network associated with this task (Hartwigsen, 2018), and it is possible that, when activity of the PMd is suppressed, the PMd on the other side support the suppressed one (O’Shea et al., 2007). In the present study, taking this point into consideration, the conventional single tSMS as well as the new triple tSMS were used to stimulate not only the unilateral motor association cortex (MAC) including the PMd (Kirimoto et al., 2011), but also the bilateral MAC.

Accordingly, the purpose of the present study was to investigate the effect of tSMS over the unilateral or bilateral MAC on performance of RT tasks. Since the effect of tSMS has been revealed to depend on task difficulty (Gonzalez-Rosa et al., 2015; Chen et al., 2021; Watanabe et al., 2021, 2023), we adopted simple and choice reaction time (SRT and CRT) tasks, as the CRT task, requiring additional visual attention and cognitive resources to select the effector, is considered more difficult than the SRT task. We hypothesized that tSMS over the MAC would influence the RT performance particularly when the triple tSMS was applied over the bilateral MAC during the CRT task.

## 2 Materials and methods

### 2.1 Participants

Eighteen healthy adults (10 female, mean age ± SD = 23.9 ± 3.8 years) participated in Experiment 1, and fifteen healthy adults (4 female, 23.4 ± 3.7 years) participated in Experiment 2. Six of them participated in both experiments. All participants provided written informed consent prior to the experiment, which was conducted in accordance with the principles of the Declaration of Helsinki. All participants in Experiment 1 (mean Laterality Quotient ± SD = 96.1 ± 7.78) and 2 (mean Laterality Quotient ± SD = 91.2 ± 10.3) were right-hand dominant according to the Edinburgh Handedness Inventory (Oldfield, 1971), and had normal or corrected-to-normal vision. This study was approved by the ethics committee of Hiroshima University (No. C-332).

### 2.2 Procedure

**FIGURE 1:**
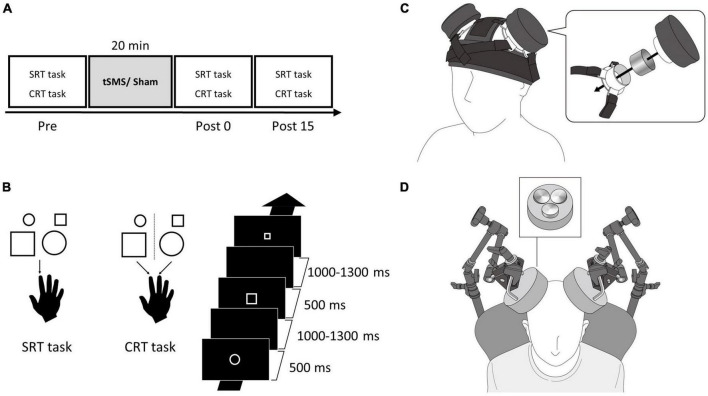
Single and triple tSMS setup and experimental protocol. **(A)** Participants performed SRT and CRT tasks before (Pre), immediately after (Post 0), and 15 min after (Post 15) tSMS or sham for 20 min. **(B)** In the SRT task, participants pressed a button with their right index finger in response to all the figures. In the CRT task, participants pressed a button with their right index finger in response to a small circle or large square and pressed a button with their right middle finger in response to a large circle or small square. The visual stimuli were displayed for 500 ms with an interstimulus interval of 1,000–1,300 ms. **(C)** In Experiment 1, a magnet and a non-magnetic stainless-steel cylinder (sham) were placed on the MAC using the custom headgear. This image is adapted from a previous study under Creative Commons Attribution (CC BY) license (Chen et al., 2021). **(D)** In Experiment 2, triple tSMS (or sham) was held using an arm type lighting stand. CRT, choice reaction time; MAC, motor association cortex; SRT, simple reaction time; tSMS, transcranial static magnetic stimulation.

Participants were seated in a comfortable chair with armrests and a mounted headrest in a dark room. They faced a 27-inch monitor (LCD-MF276XDB, I-O DATA, Japan) placed at a distance of 150 cm. The location of the PMd was determined using TMS, which was delivered using a figure-of-eight coil (external loop diameter of 95 mm) connected to a stimulator (Magstim 200, Magstim, UK). The motor cortex site where TMS consistently evoked visible twitch of the first dorsal interosseous muscle was determined as the motor hotspot (Varnava et al., 2011). The PMd was defined as 2 cm anterior to the hotspot (Fink et al., 1997; Gangitano et al., 2008), and its location was marked on the scalp with a pen. Prior to the experimental session, participants practiced SRT and CRT tasks by performing three blocks of 60 trials (a total of 180 trials) for each task. Then, they performed the tasks (three blocks of 60 trials for each task) in a random order before (Pre), immediately after (Post 0), and 15 min after the tSMS or sham stimulation (Post 15) (Figure 1A). Participants were blinded to the stimulation condition, and, after the experiment, they were asked which stimulation they think they have received in order to confirm whether blinding was successful or not.

### 2.3 Simple and choice reaction time tasks

The visual stimuli used in the SRT and CRT tasks included four types of figures: small circle (diameter, 2.6 cm), large circle (diameter, 5.3 cm), small square (side, 2.3 cm), and large square (side, 4.6 cm). All visual stimuli were presented in the center of monitor and in white color on a black background. The visual stimuli were displayed for 500 ms with a random interstimulus interval of 1,000–1,300 ms. Participants placed their index and middle fingers on two separate buttons on a custom-made device. In the SRT task, they pressed the button with their right index finger in response to all the figures (Figure 1B). In the CRT task, they pressed the button with their index finger in response to a small circle or large square and pressed the button with their middle finger in response to a large circle or small square (Figure 1B). The instruction was to press the button as quickly as possible when the visual stimulus was presented. The visual stimuli were presented using a customized LabVIEW program (National Instruments, Austin, TX, USA).

### 2.4 Transcranial static magnetic stimulation over the MAC

In Experiment 1, we applied the conventional single tSMS using a cylindrical NdFeB magnet (diameter, 50 mm; height, 30 mm) with a surface magnetic flux density of 534 mT, maximum energy density of 49 MGOe, and strength of 862 N (88 kgf) (NeoMag, Ichikawa, Japan). A non-magnetic stainless-steel cylinder of the same size, weight, and appearance was used for sham stimulation (NeoMag, Ichikawa, Japan). The center of the magnet or stainless-steel cylinder was placed on the mark on the scalp (PMd) using custom-made headgear (Hiroshima Prefectural Technology Research Institute and Fashion Reform Ace, Hiroshima, Japan) (Chen et al., 2021; Figure 1C). Participants received each of the following stimulations for 20 min: (1) tSMS over the left MAC (unilateral), (2) tSMS over the bilateral MAC (bilateral), and (3) sham stimulation over the bilateral MAC (sham). For the unilateral stimulation, the stainless-steel cylinder was placed on the right MAC as well. During the tSMS or sham stimulation, participants watched a silent movie to avoid falling asleep. Three stimulation conditions were randomized among the participants. Each stimulation was conducted on separate days (at least 3 days apart) at similar hours of the day to avoid carryover effects.

In Experiment 2, we used a triple tSMS system with three NdFeB magnets placed close to each other (New-Mag, Sakura, Japan). The north pole of the three magnets were embedded in a foundation made of non-magnetic material (a diameter of 140 mm) (Figure 1D). These magnets had the same flux density, maximum energy density, and strength as the magnet used in the conventional single tSMS. Sham stimulation was applied using a device with three non-magnetic stainless-steel cylinders embedded in the foundation. Its size and appearance were same as the triple tSMS system. Triple tSMS or sham device was held using an arm type lighting stand (Avenger C-stand, Manfrotto, Cassola, Italy), and the center of the foundation was localized just above the mark (PMd). The following procedure was same as the Experiment 1. Details of triple tSMS system are described elsewhere (Shibata et al., 2022).

### 2.5 Simplified simulation of the spatial distribution of the magnetic field

We compared the distributions of magnetic field on the human cortical surface generated by single and triple tSMS placed above the bilateral MAC. The simulation was conducted in COMSOL Multiphysics v6.0 (COMSOL, Burlington, MA, USA) (Shibata et al., 2022). ICBM152 (Fonov et al., 2009, 2011) was used for a human head model. In simulation, the head was surrounded by an air sphere of radius 40 cm. To simplify the simulation process, the layers of skin, skull, and cerebrospinal fluid and those of gray matter and white matter were merged into the outer and inner layer, respectively.

### 2.6 Data and statistical analysis

Reaction time (RT) was defined as the interval between the onset of visual stimulus and the button press. Responses faster than 150 ms or slower than the mean + 3SD and those with choice errors were excluded from the analysis (Hultsch et al., 2002; Berger and Kiefer, 2021). Consequently, 4.41 and 4.17% of data were excluded for SRT and CRT tasks, respectively, in Experiment 1, and 4.23 and 4.54% of data for SRT and CRT tasks, respectively, in Experiment 2. We evaluated the mean RT, SD of RT, and accuracy. The data at Post 0 and Post 15 were normalized to that at Pre. Normality of data were checked using Shapiro–Wilk test, and the data with non-normal distributions were log transformed [log(x + 1)]. Two-way repeated-measures analyses of variance (ANOVA) were conducted to examine the effect of tSMS over the MAC on the task performance, with Stimulation (Sham, Unilateral, and Bilateral) and Time (Pre, Post 0, and Post 15) as factors. Bonferroni’s correction for multiple comparisons was used for *post hoc* analysis. We used the Fisher’s exact test to assess whether participants were blinded to stimulation conditions. The level of significance was set at *p* < 0.05. All statistical analyses were conducted using SPSS (IBM, Armonk, NY, USA) and R (R Development Core Team).

## 3 Results

### 3.1 Experiment 1: Effect of single tSMS over the MAC on RT performance

None of the participants reported any adverse effects during or after single tSMS. There was no association between actual stimulation condition and participant’s judgment (Fisher’s exact test, *p* = 0.138; Table 1), demonstrating that participants were unable to determine the stimulation condition.

**TABLE 1:** Participants’ judgements on the stimulation conditions of single tSMS.

	Actual stimulated conditions			
	**Sham**	**Unilateral**	**Bilateral**	**Total**
**Participant’s judgements**				
Real	1	4	4	9
Sham	3	0	0	3
Cannot say	14	14	14	42
Total	18	18	18	54

**FIGURE 2:**
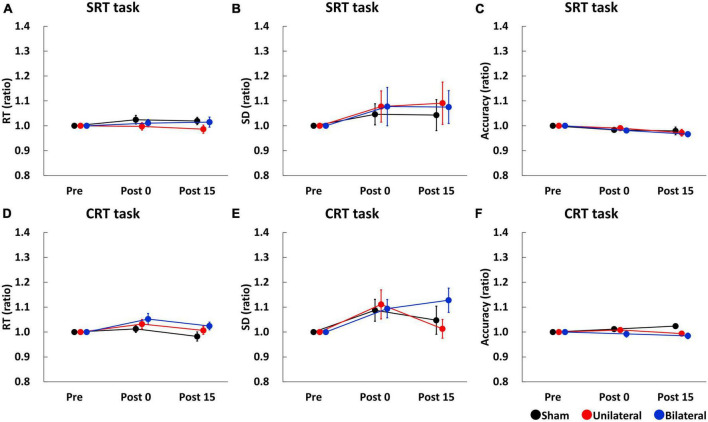
Serial changes in the average of RT **(A,D)**, SD **(B,E)**, and accuracy **(C,F)** before (Pre), immediately after (Post 0), and 15 min (Post 15) after single tSMS/Sham. Single tSMS did not affect the performance of SRT or CRT tasks. Black, red, and blue lines indicate results from Sham, Unilateral, and Bilateral stimulation, respectively. Note that data at Post 0 and Post 15 were normalized to that at baseline (Pre). CRT, choice reaction time; SD, standard deviation; SRT, simple reaction time.

Simple reaction time (SRT), SD of SRT, and accuracy of SRT task before stimulation were comparable between the stimulation conditions (SRT: mean RT ± SE = 238.57 ± 6.39 ms for Sham, 244.37 ± 7.12 ms for Unilateral, and 240.76 ± 6.92 ms for Bilateral; SD of SRT: mean ± SE = 35.14 ± 2.52 ms for Sham, 41.76 ± 3.88 ms for Unilateral, and 36.90 ± 3.84 ms for Bilateral; Accuracy: mean accuracy ± SE = 96.11 ± 0.62% for Sham, 96.76 ± 0.46% for Unilateral, and 98.06 ± 0.28% for Bilateral). Figures 2A–C show SRT, SD of SRT, and accuracy of SRT task, respectively. A two-way repeated-measures ANOVA for SRT and SD of SRT indicated no significant main effect of Stimulation (SRT: F_2,34_ = 1.338, *p* = 0.276; SD of SRT: F_2,34_ = 0.071, *p* = 0.932), Time (SRT: F_2,34_ = 0.857, *p* = 0.434; SD of SRT: F_2,34_ = 1.161, *p* = 0.325), or their interaction (SRT: F_4,68_ = 0.737, *p* = 0.570; SD of SRT: F_4,68_ = 0.046, *p* = 0.996). A two-way repeated-measures ANOVA for accuracy of SRT task revealed a significant main effect of Time (F_2,34_ = 5.895, *p* = 0.006), but there was no significant main effect of Stimulation (F_2,34_ = 0.338, *p* = 0.715) or interaction between Time and Stimulation (F_4,68_ = 0.464, *p* = 0.647).

Choice reaction time (CRT), SD of CRT, and accuracy of CRT task before stimulation were comparable between the stimulation conditions (CRT: mean RT ± SE = 460.51 ± 13.44 ms for Sham, 454.28 ± 13.42 ms for Unilateral, and 449.17 ± 13.57 ms for Bilateral; SD of CRT: mean ± SE = 111.94 ± 5.13 ms for Sham, 113.24 ± 7.47 ms for Unilateral, and 108.31 ± 5.11 ms for Bilateral: Accuracy: mean accuracy ± SE = 94.72 ± 1.04% for Sham, 95.83 ± 0.81% for Unilateral, and 96.67 ± 0.82% for Bilateral). Figures 2D–F show CRT, SD of CRT, and accuracy of CRT task, respectively. A two-way repeated-measures ANOVA for CRT and SD of CRT revealed a significant main effect of Time (CRT: F_2,34_ = 5.846, *p* = 0.007; SD of CRT: F_2,34_ = 6.345, *p* = 0.005), but there was no main effect of Stimulation (CRT: F_2,34_ = 1.434, *p* = 0.253; SD of CRT: F_2,34_ = 0.729, *p* = 0.490) or interaction between Time and Stimulation (CRT: F_4,68_ = 0.941, *p* = 0.446; SD of CRT: F_4,68_ = 1.367, *p* = 0.266). A two-way repeated-measures ANOVA for accuracy of CRT task showed no significant main effect of stimulation (F_2,34_ = 2.064, *p* = 0.143), time (F_2,34_ = 0.230, *p* = 0.718), or their interaction (F_4,68_ = 2.388, *p* = 0.092).

### 3.2 Experiment 2: Effect of triple tSMS over the MAC on RT performance

Similar to single tSMS, none of the participants reported any adverse effects during or after triple tSMS. There was no association between actual stimulation condition and participant’s judgment (Fisher’s exact test, *p* = 0.903; Table 2). This indicates that participants were unable to determine the stimulation condition.

**TABLE 2:** Participants’ judgements on the stimulation conditions of triple tSMS.

	Actual stimulated conditions			
	**Sham**	**Unilateral**	**Bilateral**	**Total**
**Participant’s judgements**				
Real	7	5	5	17
Sham	2	4	4	10
Cannot say	6	6	6	18
Total	15	15	15	45

**FIGURE 3:**
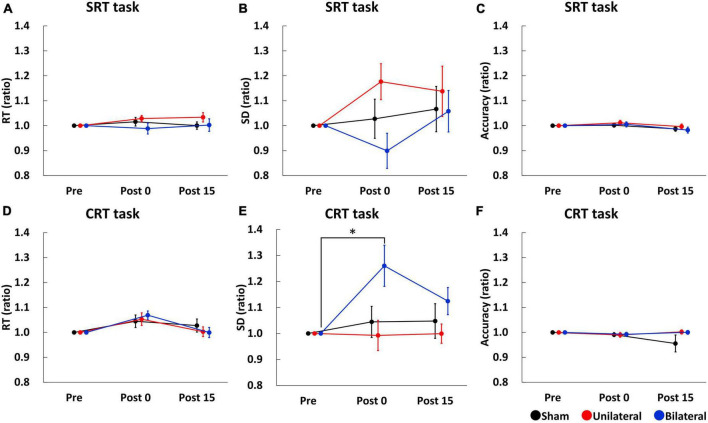
Serial changes in the average of RT **(A,D)**, SD **(B,E)**, and accuracy **(C,F)** before (Pre), immediately after (Post 0), and 15 min (Post 15) after triple tSMS/Sham. SD of CRT was significantly larger at Post 0 as compared to Pre when triple tSMS was applied to the bilateral MAC **(E)**. Black, red, and blue lines indicate results from Sham, Unilateral, and Bilateral stimulation, respectively. Note that data at Post 0 and Post 15 were normalized to that at baseline (Pre). *p = 0.01. CRT, choice reaction time; MAC, motor association cortex; RT, reaction time; SD, standard deviation; SRT, simple reaction time; tSMS, transcranial static magnetic stimulation.

Simple reaction time (SRT), SD of SRT, and accuracy of SRT task before stimulation were comparable between the stimulation conditions (SRT: mean RT ± SE = 228.58 ± 6.46 ms for Sham, 232.48 ± 7.24 ms for Unilateral, and 234.52 ± 6.82 ms for Bilateral; SD of SRT: mean ± SE = 33.25 ± 2.73 ms for Sham, 34.40 ± 3.41 ms for Unilateral, and 35.63 ± 3.32 ms for Bilateral; Accuracy: mean accuracy ± SE = 95.89 ± 0.89% for Sham, 96.22 ± 0.90% for Unilateral, and 96.78 ± 0.62% for Bilateral). Figures 3A–C show SRT, SD of SRT, and accuracy of SRT task. A two-way repeated-measures ANOVA for SRT and SD of SRT showed no significant main effect of Stimulation (SRT: F_2,34_ = 1.210, *p* = 0.313; SD of SRT: F_2,34_ = 1.526, *p* = 0.235), Time (SRT: F_2,34_ = 1.556, *p* = 0.229; SD of SRT: F_2,34_ = 0.890, *p* = 0.422), or their interaction (SRT: F_4,68_ = 0.767, *p* = 0.551; SD of SRT F_4,68_ = 1.759, *p* = 0.150). A two-way repeated-measures ANOVA for accuracy of SRT task revealed a significant main effect of Time (F_2,34_ = 5.851, *p* = 0.017), but there was no significant main effect of Stimulation (F_2,34_ = 0.620, *p* = 0.545) or interaction between Time and Stimulation (F_4,68_ = 0.824, *p* = 0.516).

Choice reaction time (CRT), SD of CRT, and accuracy of CRT task before stimulation were comparable between the stimulation conditions (CRT: mean RT ± SE = 440.33 ± 25.55 ms for Sham, 438.35 ± 16.75 ms for Unilateral, and 448.40 ± 16.55 ms for Bilateral; SD of CRT: mean ± SE = 115.83 ± 9.23 ms for Sham, 122.27 ± 9.75 ms for Unilateral, and 109 ± 7.76 ms for Bilateral; Accuracy: mean accuracy ± SE = 96.56 ± 0.79% for Sham, 96.67 ± 0.81% for Unilateral, and 96.00 ± 0.72% for Bilateral). Figures 3D–F show CRT, SD of CRT, and accuracy of CRT task, respectively. A two-way repeated-measures ANOVA for CRT revealed a significant main effect of Time (F_2,34_ = 8.279, *p* = 0.002), but there was no significant main effect of Stimulation (F_2,34_ = 0.084, *p* = 0.920) or interaction between Time and Stimulation (F_4,68_ = 1.242, *p* = 0.304). A two-way repeated measures ANOVA for SD of CRT revealed significant main effects of Stimulation (F_2,34_ = 4.715, *p* = 0.017) and Time (F_2,34_ = 3.460, *p* = 0.045), and their interaction (F_4,68_ = 2.793, *p* = 0.035). *Post-hoc* tests revealed that SD of CRT was significantly larger at Post 0 as compared to Pre in the bilateral condition (*p* = 0.01) (Figure 3E). A two-way repeated-measures ANOVA for accuracy of CRT task revealed no significant main effect of Stimulation (F_2,34_ = 1.141, *p* = 0.313), Time (F_2,34_ = 0.660, *p* = 0.454) or their interaction (F_4,68_ = 1.325, *p* = 0.277).

### 3.3 Spatial distribution of magnetic field by tSMS

**FIGURE 4:**
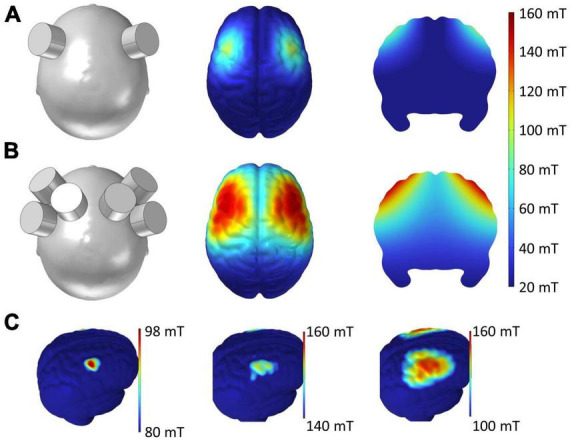
Simulated magnetic field by single and triple tSMS over the MAC. **(A)** Single tSMS. **(B)** Triple tSMS. Distribution of the magnetic field on the cortical surface is presented in the middle column. Distribution of the magnetic field on the brain slice is presented in the right column. **(C)** With single tSMS (left), the strength of magnetic field ranged from 80 to 98 mT, and its distribution was centered around the PMd (80–100 mT) with some reaching the M1 and anterior part of the PM (< 80 mT). With triple tSMS (middle and right), the strength of magnetic field ranged from 100 to 160 mT, and its distribution was centered around the PMd and M1 (> 100 mT) with some reaching the SMA, PFC, and sensorimotor cortices. MAC, motor association cortex; M1, primary motor cortex; PFC, prefrontal cortex; PMd, dorsal premotor cortex; SMA, supplementary motor area; tSMS, transcranial static magnetic stimulation.

Figure 4 shows the spatial distribution of the magnetic field by single (Figure 4A) and triple (Figure 4B) tSMS over the bilateral MAC generated in a human brain model (ICBM152). In single tSMS, the spatial distribution of the induced magnetic field was centered around the PMd (80–100 mT) (Baumer et al., 2009), with some reaching the motor cortex and a portion of the anterior part of PM (aPM) (< 80 mT) (Civardi et al., 2001). On the other hand, in triple tSMS, there was a strong magnetic field (> 100 mT) not only in the PMd but also in the sensorimotor cortices and the other MAC, such as the supplementary motor area (SMA), with some reaching the prefrontal cortex (PFC).

## 4 Discussion

In this study, for the first time, not only the conventional single tSMS but also the triple tSMS that generates a quite high magnetic field was applied to the unilateral or bilateral MAC in humans to investigate their effects on RT performance. As a result, performance of CRT task was impaired immediately after triple tSMS over the bilateral MAC. On the other hand, neither single tSMS over the unilateral/bilateral MAC nor triple tSMS over the unilateral MAC had influenced the performance of RT tasks. The simulation results revealed that triple tSMS generated a strong magnetic field over the sensorimotor areas, PFC and MAC. No adverse effects were observed under any stimulation condition, including the tripe tSMS over the bilateral MAC. The reliability of sham stimulation was confirmed to be high as well.

Although the exact mechanism of how SMFs influence the central nervous system remains unclear, some hypotheses have been proposed at a cellar level (Albuquerque et al., 2016). It has been suggested that SMFs induce reorientation of membrane phospholipids via diamagnetic anisotropy, consequently deforming the embedded ion channels, thereby altering their functions (Rosen, 2003). In addition, the magnetic field gradient produced by SMFs can induce surface tensions altering substantially the gating probability of mechanosensitive channels (Hernando et al., 2020). Meanwhile, studies in humans showed that the primary motor cortex (M1) excitability can be reduced by single as well as triple tSMS, and that the strength and range of SMFs produced by triple tSMS was greater than those by single tSMS (Shibata et al., 2022). Thus, it is reasonable to assume that triple tSMS reduced the excitability of the MAC including the PMd more strongly than single tSMS in this study.

The present study found no significant changes in RT after single or triple tSMS for both SRT and CRT tasks. Some previous studies in which LF-rTMS or cTBS was applied to the unilateral PMd reported that RT was prolonged transiently after the stimulation (Mochizuki et al., 2005; Gorbet and Staines, 2011), while the others reported no significant changes in RT (O’Shea et al., 2007; Ward et al., 2010; Lu et al., 2012). Regardless of the unilateral or bilateral tSMS, our results were consistent with the latter studies. The underlying reason behind this difference is currently unclear, but one possibility relates to compensatory activation of the non-stimulated brain regions. For example, O’Shea et al. (2007) demonstrated that LF-rTMS over the left PMd resulted in a compensatory increase in the right PMd activity, and that TMS to the right PMd showing the compensatory increase in activity prolonged CRT. To suppress this compensatory activation, tSMS was applied to the bilateral MAC simultaneously in this study; however, no significant changes in RT was observed. As the other brain regions, such as the bilateral parietal cortices, are activated during the CRT task (Johansen-Berg et al., 2002; Chouinard and Paus, 2006; O’Shea et al., 2007), it is possible that these brain regions have increased their activity to compensate for the PMd in the present study.

In contrast to the RT, SD of CRT increased immediately after triple tSMS over the bilateral MAC. This result is similar to a previous study demonstrating that cTBS over the PMd affected performance of CRT task but not of SRT task (Mochizuki et al., 2005). Observation of the effect of tSMS only on the SD of CRT and not on the SRT, SD of SRT, or CRT can be due to the sensitivity of the variables and/or cognitive load of the task. RT reflects speed of information processing, while SD of RT reflects consistency in processing speed (Jensen, 1992), suggesting that alertness and sensory processing were inconsistent across trials after triple tSMS over the bilateral MAC. Also, SD of RT has been reported to be more sensitive than mean RT as a marker of cognitive impairment (Klein et al., 2006; Schulz-Zhecheva et al., 2023). Moreover, Gonzalez-Rosa et al. (2015) demonstrated that visual search RT was prolonged after tSMS over the occipital cortex only when the task was difficult. Thus, the effect of tSMS might have been apparent only for the sensitive variable during the CRT task that is considered to be more difficult than SRT task. Another possibility can be changes in finger movement. Specifically, triple tSMS over the bilateral MAC (potentially affecting the broad areas of the brain) might have decreased the finger dexterity.

The decline in RT performance was observed only after triple tSMS and not after single tSMS. This finding could be ascribed to a stronger stimulation of the PMd and/or stimulation of multiple brain regions by triple tSMS. Indeed, the simulation results of the present study revealed that triple tSMS generated a stronger SMF in the PMd compared to single tSMS, and also that a SMF generated by triple tSMS reached to multiple brain regions. In addition, Terao et al. (2005) reported that single-pulse TMS applied over various brain regions, including the prefrontal, motor association and parietal cortices, during a pre-cued CRT task prolonged RT. Similarly, LF-rTMS over these brain regions has been found to induce a delay in RT in the same task (Terao et al., 2007). Moreover, there is a study demonstrating that patients with lesions in the PFC have greater SD of SRT and CRT than patients with non-frontal lesions or healthy controls (Stuss et al., 2003), suggesting that increased behavioral variability can be linked to the frontal brain regions (MacDonald et al., 2006). Hence, it is quite likely that our finding was attributed to the stimulation of multiple cortical regions by triple tSMS. Meanwhile, combined rTMS and fMRI study reported that rTMS over the PMd did not alter neural activity when stimulation was delivered at a strength of motor threshold (Kemna and Gembris, 2003), indicating that strength of stimulation needs to be quite high to modulate the PMd activity. Nonetheless, the neurophysiological impact of triple tSMS on the cortical activity and behavioral performance requires further investigations.

Our study has three main limitations. First, we did not assess activity of the MAC or connectivity between the brain regions. Since brain activity/connectivity can be modulated by tSMS (Gonzalez-Rosa et al., 2015; Chen et al., 2021; Shibata et al., 2021; Watanabe et al., 2023), future studies should consider this aspect. Second, accuracy of SRT and CRT tasks declined as experiment progressed. It is possible that fatigue and lapse of attention influenced our results because the declines were observed in all stimulation conditions (Williams et al., 2005; Langner et al., 2010). Third, we did not use an MRI-based neuronavigation system to identify the location of the PMd. Similar to most previous studies, we defined the location of the PMd based on the motor hotspot within the M1 (Schlaghecken et al., 2003; Mochizuki et al., 2005; Gangitano et al., 2008).

## 5 Conclusion

Single tSMS over the unilateral or bilateral MAC did not affect performance of RT tasks, whereas triple tSMS over the bilateral MAC but not over the unilateral MAC increased variability of CRT. These results suggest that RT task performance can be modulated using triple tSMS.

## Data availability statement

The raw data supporting the conclusions of this article will be made available by the authors, without undue reservation.

## Ethics statement

The studies involving humans were approved by the Ethics Committee of Hiroshima University. The studies were conducted in accordance with the local legislation and institutional requirements. The participants provided their written informed consent to participate in this study.

## Author contributions

TMa: Formal analysis, Funding acquisition, Investigation, Visualization, Writing – original draft. TW: Funding acquisition, Methodology, Software, Supervision, Writing – review & editing. KI: Data curation, Investigation, Resources, Writing – review & editing. TH: Funding acquisition, Investigation, Resources, Writing – review & editing. SS: Funding acquisition, Software, Visualization, Writing – review & editing. HKu: Writing – review & editing. TS: Writing – review & editing. TMi: Funding acquisition, Project administration, Supervision, Writing – review & editing. HKi: Conceptualization, Funding acquisition, Methodology, Supervision, Writing – review & editing.
